# Transforming the Landscape of Drug Innovation in AI Era: AlphaFold 3 and Beyond

**DOI:** 10.1002/exp2.70236

**Published:** 2026-10-11

**Authors:** Songlin He, Liangbin Zhou, Xiaoshuai Wang, Chenzhong Li, Cheng Jiang, Rocky S. Tuan, Zhong Alan Li

**Affiliations:** ^1^ Department of Biomedical Engineering, Faculty of Engineering The Chinese University of Hong Kong Hong Kong SAR China; ^2^ InnoHK Center for Neuromusculoskeletal Restorative Medicine Hong Kong Science and Technology Park Hong Kong SAR China; ^3^ Department of Rehabilitation Medicine The Third Affiliated Hospital Southern Medical University Guangzhou Guangdong China; ^4^ School of Medicine The Chinese University of Hong Kong Shenzhen China; ^5^ Institute For Tissue Engineering and Regenerative Medicine, School of Biomedical Sciences The Chinese University of Hong Kong Hong Kong SAR China; ^6^ Peter Hung Pain Research Institute, Faculty of Medicine The Chinese University of Hong Kong Hong Kong SAR China

**Keywords:** AlphaFold 3, artificial intelligence, drug discovery and development, protein structure prediction, personalized medicine

## Abstract

The traditional drug discovery and development (DDD) pipeline has long been characterized by inefficiency and high costs. The rapid evolution of artificial intelligence (AI), especially AI‐based toolkits for protein structure and interaction prediction, exemplified by AlphaFold 3 (AF3), promises to reshape the landscape of drug innovation. AF3 advances structural biology by predicting protein structures and their interactions with a range of biomolecules, including nucleic acids, small‐molecule ligands, and ions, potentially changing the conventional DDD paradigm, although prediction accuracy varies substantially across these categories. This perspective examines the impact of AF3 on drug target identification and validation, hit identification, lead optimization, and personalized clinical decision‐making, while addressing the technical challenges, ethical considerations, and governance implications. Solutions to overcome current limitations are proposed to advance the development of novel therapeutics.

## Graphical abstract

Traditional drug development is time‐consuming and costly, and new tools are urgently needed to improve efficiency. This perspective reviews the considerable potential of AlphaFold 3 as a next‐generation predictive tool for drug discovery, particularly in target identification and validation, hit identification and lead optimization, and personalized clinical decision‐making. Among these, its applications in personalized medicine are the least mature and currently remain largely at the research stage, given their dependence on patient‐specific data and clinical validation. As its use and development advance, technical limitations in structural prediction reliability and the associated ethical and regulatory concerns must also be addressed.

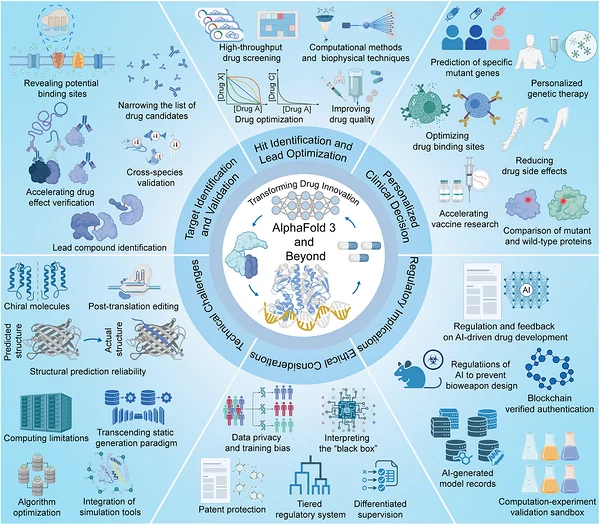

## 1 General Background of AlphaFold 3

Developed jointly by Google DeepMind and Isomorphic Labs, AlphaFold 3 (AF3) was announced on May 8, 2024. Compared with earlier versions, AF3 extends structure prediction beyond individual proteins to complexes involving proteins, DNA, RNA, and other molecular components [1, 2]. This expanded capability may support applications in molecular biology and drug discovery. Although AF3 represents a significant advance, its impact varies markedly across prediction categories. For monomeric protein prediction, the improvement in core accuracy over AlphaFold 2 (AF2) has entered a stage of diminishing returns. On standard benchmarks, AF3's global prediction confidence may elevate the average predicted local distance difference test (pLDDT) score from AF2's approximately 92 to 94—a marginal gain, as both scores already approach the intrinsic error range of medium‐resolution experimental structures [1]. Consequently, for most soluble globular proteins, predictions from either model serve as a highly reliable structural starting point. The true generational leap, however, lies in protein‐ligand complex prediction. Unlike AF2, which cannot predict ligands, and traditional docking tools like AutoDock Vina that require a predefined, rigid binding pocket, AF3 can generate plausible binding pocket geometry and ligand poses for novel targets. Comparative tests indicate that this capability offers a substantial advantage in scenarios where the binding site is unknown. Nevertheless, for local ligand pose refinement within a well‐ defined precise pocket, specialized docking programs may retain an advantage in computational speed. Furthermore, AF3 demonstrates marked improvement in predicting the assembly of large macromolecular complexes. It achieves this through a more accurate Predicted Alignment Error (PAE) map, which better informs the relative orientation between subunits. For instance, where AF2 might mispredict the docking orientation in an antibody‐antigen complex, AF3's PAE map more reliably delineates the true interface. This advancement is quantitatively reflected in significantly higher interface DockQ scores for AF3 compared to its predecessor, indicating superior model quality for biological assemblies.

AF3 pioneered a precise structural prediction framework upon which reliable models for many subfields can be developed. Recently, David Baker's team developed RFdiffusion 3 based on the AF3 model, successfully designing and experimentally characterizing a cysteine hydrolase [2]. The activity of the artificially designed cysteine hydrolase exceeds that of existing hydrolases. Mao's team utilized AF3 to design and characterize stable triangular DNA nanostructures that do not naturally occur. These all demonstrate the enormous potential of AF3 in the design of new drugs [3]. It can predict protein structures in a fraction of the time required by traditional laborious experimental methods such as X‐ray crystallography or cryo‐electron microscopy. With the ability to predict these structures with unprecedented accuracy and unparalleled speed, AF3 has the potential to revolutionize drug design and development (Figure 1) [4]. Thus, it can significantly shorten the DDD timeline, allowing researchers to rapidly identify and optimize drug candidates, particularly when combined with advanced in vitro disease modeling techniques like organoids and organs‐on‐chips [5, 6].

**FIGURE 1:**
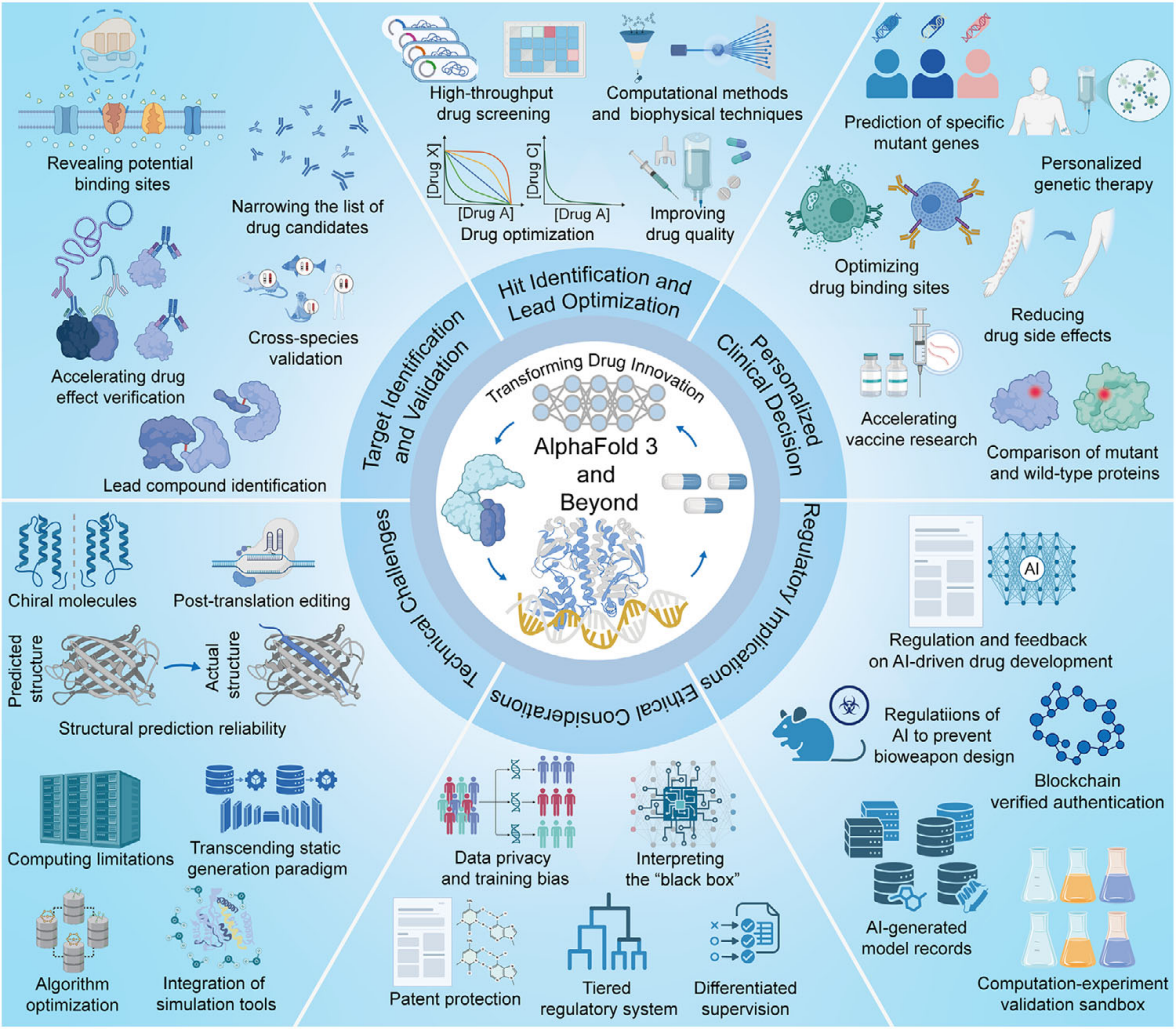
Potential applications and limitations of AlphaFold 3 (AF3) in drug discovery. AF3 predicts the structures of biomolecular complexes, providing structural insights that may support target assessment, hit identification, and lead optimization. These predictions require experimental validation. Potential applications in personalized medicine remain investigational, and AF3 alone does not provide patient‐specific treatment recommendations. Technical limitations and ethical and regulatory considerations must be addressed when translating these applications into practice (created in BioRender. https://BioRender.com/32oyhym).

## 2 AlphaFold 3's Roles in Target Identification and Validation

Target identification and validation are two crucial steps in the drug discovery process. The primary goal of target identification is to identify biological targets, typically proteins, that are involved in a disease process. AF3's ability to predict protein structures from amino acid sequences opens up new possibilities for identifying previously unknown drug targets [7]. Compared to the method of large‐scale screening through library construction, AF3 can predict the secondary and tertiary structures of proteins, allowing researchers to design drugs in a targeted manner. AF3's structural model is able to simulate dynamic protein behaviors, such as protein conformational changes and interactions with nucleic acid molecules. Many diseases, including cancer and neurodegenerative disorders, are associated with proteins that are difficult to study due to the lack of structural information. Due to the complexity of protein dynamics and the interference of various factors during their interactions, it is almost impossible to characterize all proteins using cryo‐electron microscopy observations. This poses considerable difficulties for the development of biomolecular drugs using protein targets. AF3 can predict the structures of most proteins, provide detailed maps of these proteins, and reveal potential binding sites for therapeutic intervention. AF3 cannot replace a deep understanding of biological mechanisms and pathway dynamics, which are the cornerstone of target identification. However, its revolutionary role lies in transforming this understanding from abstract sequence or functional information into a concrete, visual, and computable three‐dimensional (3D) structural language, thereby providing unprecedented physicochemical insights for critical decisions in target identification.

AF3 can also support target validation, the stage at which candidate targets are confirmed to be causally involved in disease and amenable to therapeutic modulation [8]. Validation ultimately depends on experimental evidence, including genetic knockdown or knockout, overexpression, pharmacological or antibody‐based modulation, and studies in animal models and patient samples. AF3 complements rather than replaces these experiments. By predicting protein–ligand complex structures, it generates testable hypotheses about binding modes and candidate binding sites, which help prioritize compounds and mutations for experimental testing. This is also relevant when validation relies on animal models, since structural differences between human proteins and their orthologs can limit how well results translate. Comparing predicted structures across species allows researchers to assess binding‐site conservation before selecting a model organism. In addition, AF3 can explicitly model certain post‐translational modifications, such as phosphorylation and glycosylation, making it possible to examine how these modifications affect local conformation or ligand accessibility, information that can guide the optimization of potency and selectivity. Nevertheless, AF3 outputs are static structures of varying confidence and should be interpreted together with their confidence metrics (e.g., pLDDT, PAE) and experimental data.

## 3 AlphaFold 3's Roles in Hit Identification and Lead Optimization

The primary goal of hit and lead identification is to find compounds that have the potential to interact effectively with the biological target, with hits being confirmed before they are advanced as leads. Accurate protein structure prediction is essential for rational drug design. The development strategies for lead compounds primarily encompass: 1) high‐throughput screening, which involves evaluating extensive compound libraries to identify those with activity against the target; 2) computational methods, such as virtual screening and molecular docking, which use computer simulations to predict potential lead compounds; and 3) biophysical techniques, including NMR, X‐ray crystallography, and surface plasmon resonance, which are utilized to analyze interactions between compounds and their targets. With AF3, researchers can model how small molecules or biologics interact with their target proteins at an atomic level. Currently, drug development at the protein level heavily relies on the reliability of protein structures. Given the dependence on existing protein data, it is essential to integrate molecular dynamics simulations and molecular docking methods to validate and optimize current drug candidates. However, for diseases whose relevant proteins lack experimental structures, the absence of corresponding protein data hinders theoretical drug design and development. For instance, while there are already therapeutic agents available for cancer, the target proteins differ among various cancer subtypes; identifying subtype‐specific target proteins is therefore necessary to tailor the treatment plan effectively. AF3 offers the capability to predict relevant protein structures, providing a structural‐biology foundation for drug development where experimental structures are unavailable. This information enables the design of drugs that complement their target sites, enhancing selectivity and reducing off‐target effects, although the resulting models still require experimental validation. On the other hand, AF3 can predict the structures of protein‐ligand interactions, protein complexes, and post‐translational modifications of proteins, supporting the structural evaluation of candidate compounds in virtual screening. Although AF3 currently has low prediction accuracy for glycosylation, phosphorylation, and lipidation, it can fill a large research gap in the screening of targets whose static structures are otherwise unavailable. With the development of oligonucleotide therapeutics and small‐molecule drugs, the development of more effective clinical therapeutics has become increasingly feasible. By enhancing the quality of initial drug candidates, AF3 has the potential to increase the success rate of drug development projects. This ultimately results in the identification of a list of potential lead compounds that exhibit the desired biological activities.

Lead optimization is a pivotal stage in drug development [9]. The main objective of lead optimization is to confirm that the identified leads are genuine hits and to improve their potency, selectivity, and drug‐like properties so that they can be developed into drugs. The lead optimization process includes secondary screening, Structure‐Activity Relationship (SAR) studies, pharmacokinetic studies, and preliminary toxicity testing. Secondary screening involves testing hits from the primary screening in more specific and stringent assays to confirm their activities. SAR studies explore and optimize the relationship between the chemical structure of leads and their biological activity. Pharmacokinetics examines the absorption, distribution, metabolism, and excretion (ADME) properties of lead compounds. Additionally, preliminary toxicity testing assesses the safety and potential side effects of the leads. The application of AF3 can expedite these processes by predicting the structural consequences of chemical modifications and/or altered molecular structures, thereby supporting the enhancement of drug quality. AF3 can model protein structures in different states, helping to optimize lead compounds so that they accommodate multiple states of the target protein, thereby enhancing binding efficiency and stability; because it usually predicts a single dominant conformation, these predictions should be complemented by molecular dynamics simulations. By predicting the interaction patterns between drugs and their targets, AF3 can help to identify the structural features responsible for binding and guide modifications aimed at improving metabolic and distribution properties. Furthermore, after identifying initial lead compounds, the molecular structure or functional groups can be optimized based on predictive results to enhance their drug‐likeness, while druggability—the suitability of the target itself for drug binding—should be evaluated separately. In the lead optimization phase, AF3 provides detailed atomic‐resolution information on compounds, helps analyze molecular frameworks, and informs novel molecular design strategies. Drug development typically requires simultaneous optimization of multiple parameters, including potency, selectivity, solubility, and metabolic stability. The predictive results from AF3 offer structural foundations for the target‐dependent parameters, such as potency and selectivity, while solubility and metabolic stability are determined largely by the compound itself and must be addressed with dedicated predictive models and experimental assays. Ultimately, researchers will obtain a refined set of validated lead compounds with confirmed activity and favorable drug‐like properties.

## 4 AlphaFold 3's Roles in Personalized Clinical Decisions

The insights provided by AF3 hold significant potential for advancing personalized clinical decisions [10, 11]. By predicting the 3D structures of proteins carrying patient‐specific variants, researchers can generate hypotheses about the molecular mechanisms through which these alterations influence drug binding and therapeutic efficacy, which must then be tested experimentally. For example, cancer patients often harbor specific genetic mutations that lead to structural changes in proteins, which may affect their functions and interactions with drugs. AF3 can predict the 3D structure of proteins impacted by such mutations, supporting the design of targeted drugs tailored to patient‐specific proteins and informing treatment selection; however, the predicted structures of point mutants often differ only subtly from the wild‐type structure, and AF3 does not estimate binding affinity, so functional consequences require experimental verification. This capability facilitates the development of personalized therapies aligned with a patient's genetic profile, potentially improving treatment outcomes and lowering the risk of adverse effects.

Moreover, certain mutations can alter the active site of a protein, affecting drug binding and efficacy. With the capability to predict these changes in proteins, AF3 enables drug developers to design new drug molecules or optimize existing ones so that they better complement specific target proteins, although the resulting binding modes and expected gains in affinity must be confirmed experimentally. When integrated with genomic data, AF3 can contribute structural information to the development of personalized cancer vaccines, for example, by modeling the mutated protein antigens, and to immunotherapy research; the antigen presentation step itself, however, lies outside its scope and requires dedicated methods. Additionally, for polymorphic metabolic enzymes and receptor variants, predicted structures can complement pharmacokinetic modeling, helping to inform dosage selection and reduce the risk of side effects, ultimately enhancing precision medicine strategies.

AF3's connection to personalized clinical decisions lies in its role as a key “translator” and “hypothesis generator,” but it must be carefully embedded within a broader multi‐omics integration and clinical decision‐making framework to realize its value. Specifically, this approach begins with genomic sequencing of a patient's tumor or tissue sample to identify unique pathogenic mutations. AF3 is then used to rapidly generate and compare high‐precision 3D structural models of the mutant and wild‐type proteins. This reveals at the atomic level whether the mutation disrupts protein folding stability, alters its interaction interface with downstream effectors, or unexpectedly creates a new drug‐targetable pocket. However, this purely structural insight alone is insufficient to guide treatment. It must be systematically integrated and computationally analyzed with the patient's pharmacogenomics data (such as CYP450 enzyme genotypes to predict drug metabolism rates), transcriptomics and proteomics background (to assess the network importance of the target in disease pathways), and available medicinal chemistry knowledge bases. The ultimate goal is to comprehensively assess the structural vulnerability of the mutation and the pharmacological feasibility of existing or potential therapies in an individual patient, thereby providing clinicians with a recommendation. This might involve, for example, matching a marketed targeted drug to the structural feature, or explicitly indicating that the patient should be enrolled in a clinical trial targeting their specific variant structure, rather than providing a single definitive answer.

## 5 Technical Challenges

While AF3 offers numerous benefits, it is essential to recognize its limitations and the challenges that remain in areas such as antigen‐antibody interactions, recognition of chemical properties (e.g., chirality), dynamic conformations, disordered protein structures, and geometric inconsistencies (Figure 1). We summarize the protein‐processing capabilities of AF3 (Table 1). It shows limited prediction capabilities for non‐structural protein domains, dynamic proteins, and proteins with low sequence homology [12]. Additionally, AF3 faces challenges in predicting multi‐subunit protein complexes [13]. For example, when predicting proteins with post‐translational modifications, such as glycosylation, phosphorylation, and lipidation, the accuracy remains relatively low. This may hinder the development of drugs targeting certain genes or proteins. AF3's training database should be regularly updated with the latest structural biology research results.

**TABLE 1:** Processing performance of AlphaFold 3.

Protein types	Processing performance of AF3
Soluble globular proteins	AF3 maintains similar accuracy to AF2 in predicting the folding of soluble proteins, especially performing better in stable regions [14, 15].
Protein‐small molecule ligand complex	The ability is significantly enhanced, enabling more accurate prediction of binding pockets and ligand poses compared to AF2 [16].
Protein‐nucleic acid complex	It performs well and can predict the interaction interface between proteins and DNA/RNA [17].
Intrinsically disordered proteins	The conformational ensemble is very weak, and AF3 cannot predict its dynamic changes [18].
Transmembrane proteins	Accuracy is uneven and, on the whole, lower than that achieved for soluble proteins [19].
Ultra‐large dynamic complex	Due to AF3's limited capabilities, it is difficult to accurately predict the overall assembly of complexes with dozens of subunits [20].
Proteins with rare folds or novel designs	Predictions for these proteins show a low confidence level [21].
Proteins that are highly dependent on post‐translational modifications or specific cofactors.	Direct prediction of their functional state structure is not feasible; further refinement with AF3 is therefore required to improve predictive accuracy [22].

As structural biology continues to advance, the subcategories of proteins will become increasingly diverse. Timely updates to the training database are crucial for predicting drug‐protein binding targets. In addition, AF3 incorporates the Pairformer and Diffusion modules to streamline its architecture, which, however, demands significant computational resources. For large‐scale experiments, such as drug screening or genomics research, that require extensive predictions, using AF3 may necessitate considerable computational power and time. Therefore, the algorithm architecture should be further optimized, and the utilization rate of GPU hardware should be improved. Additionally, integrating AF3 into existing drug discovery workflows will require significant adjustments, including the development of new computational tools and protocols (Table 2). Furthermore, AF3 still needs to be integrated with additional biological simulation tools to fully capture the entire structural biology process, from DNA to RNA to protein.

**TABLE 2:** Comparison of different calculation methods.

Method	Representative tools	Core principles	Advantages	Limitations
Deep learning [23]	AlphaFold 3	Predicting structure directly from sequence using deep learning	It has achieved near‐experimental accuracy in single‐chain protein prediction, while its speed far exceeds that of traditional methods	Its ability to predict dynamic complexes is insufficient
Traditional homology modeling [24]	SWISS‐MODEL	Modeling construction based on homologous templates with known structures	Model quality depends directly on template similarity; the relationships are transparent	Cannot be performed without a template
Physics simulation [25]	Molecular Dynamics	Conformational search and simulation based on physical force fields and statistical potential energy	It can simulate dynamic processes, calculate free energy, and assess the effects of sudden changes	Limited search capabilities, susceptibility to local optima, and dependence on the force field

The limitations of AF3 stem from the inherent constraints of its predictive paradigm. First, it essentially generates a static snapshot of the dominant conformation, failing to reproduce the dynamic processes of protein function (such as conformational changes or binding intermediates), and its predictions are detached from specific cellular environments and modifications, which are often functional determinants. Second, as a data‐driven pattern generator rather than a physical simulator, it lacks a mechanistic foundation and cannot directly calculate binding free energy or mutation effects; its atomic‐level local geometric accuracy may also fall short of molecular dynamics simulations. Third, its exceptional performance is highly dependent on training data; accuracy decreases in regions with sparse or low‐quality experimental structures (such as certain membrane proteins), and it may inherit structural biases from the data, over‐predicting over‐characterized families while lacking confidence in novel folds. Finally, the model's complex “black box” nature leads to interpretability dilemmas; when predictions are incorrect, it is difficult to trace the physical or evolutionary roots, posing additional challenges to the reliability assessment of key drug targets.

The core improvement of the AF3 architecture lies in transcending the current static generation paradigm, with close collaboration across three levels: enhancement of the diffusion module, innovation of the evaluation system, and upgrades to data and training techniques. First, in the diffusion module, by introducing environmental and functional states as conditional inputs (such as specific ligands, post‐translational modifications, and physicochemical conditions), it can transform from generating a single static conformation to predicting an ensemble of multi‐state structures dependent on the environment. Simultaneously, deeply integrating equivariant networks and lightweight physical force field constraints into the denoising network can significantly improve the local atomic precision and physical plausibility of the generated structures. Furthermore, developing a multi‐scale hierarchical diffusion framework from residues to atoms holds promise for efficiently handling ultra‐large complexes and better characterizing flexible regions. Second, to accurately evaluate these dynamic and functional outputs, the performance index system must evolve synchronously. In addition to optimizing the existing pLDDT and PAE indices, it is urgent to develop new standards for quantified conformational diversity (such as conformational clustering entropy), dynamic characteristics (such as the correlation between the predicted B factor and experiments), and physical stability (such as rapid molecular mechanics energy scoring), and ultimately integrate a module for predicting binding free energy changes (ΔΔG) that can directly serve drug design. Finally, the technical details for realizing the above‐mentioned architectural innovations depend on breakthroughs in basic supporting technologies: guiding experimental verification of highly uncertain targets through active learning strategies and jointly constructing a more balanced and comprehensive training dataset by using physical simulation to generate synthetic data to overcome existing data biases; and making this enhanced system practically feasible by optimizing training objectives (such as aligning intermediate states in the denoising process) and engineering compressed inference models.

## 6 Iterative Computational–Experimental Framework for AlphaFold 3 Applications

Despite the powerful capabilities of AF3, it is also important to bridge the gap between AI predictions and experimental validation. AF3 predictions should not be reduced to binary yes/no judgments. The inherent confidence metrics of AF3, such as the predicted local distance difference test (pLDDT) and predicted alignment error (PAE), should guide decision‐making [26]. For example, regions with high‐confidence predictions (e.g., pLDDT > 90) are designated as starting points for high‐priority experimental validation, whereas low‐confidence regions (e.g., pLDDT < 70) are explicitly flagged as “high‐risk” and recommended for earlier, more rigorous experimental scrutiny. This can provide a direct rationale for allocating wet‐lab resources. Second, a “hypothesis‐driven” iterative validation loop should be established. This framework promotes a closed‐loop, cyclical process rather than a single prediction‐verification step. This process can be implemented through experimental approaches such as site‐directed mutagenesis of predicted binding‐interface residues coupled with binding‐affinity measurements or by direct comparison with experimentally determined structures. Discrepancies between experimental results and predictions should be treated as valuable opportunities to refine models and deepen mechanistic understanding. Such discrepancies can further inform molecular‑dynamics simulations or serve as training data for fine‑tuning AI models, thereby underscoring a collaborative partnership in which computation and experiment mutually correct and synergistically advance. Finally, to systematically evaluate and manage differences between predictions and experiments, it is necessary to define quantitative standards for “consistency” and “deviation”—using metrics such as the root‑mean‑square deviation (RMSD) of key binding residues and interaction‑fingerprint similarity. It should also incorporate a “deviation‑alert” mechanism: when key metrics exceed predefined thresholds, the iterative analysis cycle is automatically triggered, ensuring that discrepancies are promptly and systematically examined.

## 7 Ethical Considerations

The adoption of AI technologies like AF3 in drug discovery also raises important ethical and regulatory issues (Figure 1). To develop personalized treatment plans that draw on AF3 predictions, a vast amount of patient data is essential. This includes genomic information, proteomic data, and other sensitive details. Ensuring the privacy and security of these data is a crucial ethical issue. Because such data are collected and governed by different institutions and jurisdictions, questions of consent, reuse, and access arise well before any structure prediction is performed, and they must be settled at the level of the overall clinical workflow rather than by the model itself. Although AF3 is a highly advanced AI tool, its predictions are shaped by the composition of its training data, which consists of experimentally determined structures rather than patient genomes. Protein families and conformational states that are poorly represented in the PDB—including many human membrane proteins and several subfamilies of therapeutic interest—are therefore predicted less reliably, and this imbalance can propagate into downstream drug design. Bias can also enter at the interpretation stage: if a variant is classified using reference datasets, genotyping panels, or electronic health records drawn mainly from one population, the resulting decision may be less accurate for patients from other populations. For example, the EGFR mutations are more common in lung cancer patients of Asian descent. Whereas the mutant protein itself is structurally well characterized, underrepresentation of Asian patients in the genomic and clinical datasets used to interpret variants and guide treatment selection could lead to less accurate prediction of treatment response and to less effective treatment plans [27].

Another issue is whether AF3‐predicted protein structures can be patented as new discoveries or inventions, especially when the structures have not been validated through traditional experimental methods. Patent‐related laws should be established to ensure the value of predictive data after extensive experimental verification. Additionally, while the protein data of AF3 is publicly available, in cases involving sensitive diseases or commercially valuable targets, a careful balance between open data sharing and intellectual property protection must be considered. In addition, the “black‐box” nature of AI systems such as AF3 remains a concern. Especially in healthcare and drug development, it is crucial to be able to interpret the predictions made by AI systems to understand the underlying logic. If AI predictions lead to negative outcomes or biases, it is essential to trace and explain the decision‐making process.

Furthermore, like many other advanced technologies, AF3 has potential dual‐use implications. Protein structure prediction can, in principle, lower the barrier to designing molecules with harmful properties, a concern that has been raised specifically for pathogen‐related biomolecules. While AF3 is intended to advance scientific research and drug development, its capabilities could also raise potential biosecurity implications. This risk was acknowledged in connection with the release: access through the public server is subject to certain limitations and terms of use, and the model weights were subsequently distributed under a non‐commercial license with prohibited‐use restrictions. Whether these safeguards are sufficient remains debated, and proposed governance measures include query screening, know‐your‐customer requirements for structural biology service providers, and clear norms for reporting concerning outputs. Dual‐use risk must also be distinguished from legitimate research on pathogen structures, which underpins vaccine and antiviral development; conflating the two would impede essential public health work. Additionally, the rapid development of AI technology may exacerbate global technological inequalities, particularly in low‐ and middle‐income countries that lack the computational resources, structural biology expertise, and experimental validation capacity needed to apply these tools—gaps that licensing terms restricting commercial use may widen further. Ensuring the responsible use of AI in research and development is paramount, which requires not only technical safeguards but also coordinated international oversight and equitable access to these methods.

To systematically address the ethical challenges associated with AI‐driven structural prediction in drug discovery, establishing a comprehensive mitigation framework that integrates technical safeguards with transparent governance is necessary. This approach can employ adversarial debiasing algorithms to proactively remove sensitive attribute information during model training, thereby promoting fairness and reducing bias. Furthermore, a federated learning paradigm can be implemented to enable training on distributed data without centralizing raw datasets, thus protecting data privacy at its technological foundation. A risk‐assessment‐based hierarchical access control system can be established to provide differentiated oversight and auditing of queries according to their risk levels, thereby managing the potential for dual‐use abuse. These technical measures must be coupled with transparent governance policies, regular ethical reviews, and the development of global norms to form a multilayered governance system that guides responsible technological development, maximizing scientific benefits while minimizing risks to fairness, privacy, and security. Responsible implementation requires regulatory agencies to establish multilayered validation frameworks addressing both technical reliability and sociodemographic equity, centered on mandatory transparency protocols that compel pharmaceutical developers to disclose AI architectures, training data sources, and validation metrics—enabling effective regulatory auditing of algorithmic decision‐making. Critical safeguards include differential privacy mechanisms for anonymizing patient genomic data, homomorphic encryption for securing metadata in cloud computations, and a rotating ethics panel comprising bioethicists, community representatives, and AI specialists to oversee clinical trial applications. Concurrently, regulatory frameworks must mandate the creation of population‐specific data repositories stratified by genetic ancestry, geographical epidemiology, and socioeconomic factors, such as encrypted sub‐databases for East Asian CYP450 variants or African HLA‐B allele distributions, to enable targeted model fine‐tuning and directly address limitations in generalizing across diverse demographics. This dual emphasis on technological rigor and representational inclusivity ensures that AI‐accelerated drug development neither compromises individual privacy nor perpetuates healthcare disparities, translating computational advances into equitable therapeutic solutions.

## 8 Regulatory Implications

To mitigate potential risks and biases in AF3's applications, researchers, funders, and journals should adopt shared guidelines covering where predictions are acceptable as evidence, how confidence metrics such as pLDDT and PAE should be reported alongside any structure used in decision‐making, and how disputed or anomalous outputs are escalated. These measures should be embedded in existing research integrity and clinical governance procedures so that responsibility for acting on a prediction is assigned to a named party rather than to the model. In addition to these general safeguards, AF3 requires measures specific to structure prediction itself, for example, routine validation of predicted binding sites against experimental structures or orthogonal computational methods, periodic audits of which protein families and conformational states are underrepresented in the training data, and documented reporting of prediction failures in retrospective studies. Without such AF3‐specific protocols, general guidance on responsible AI use remains too abstract to change day‐to‐day practice.

More specifically, algorithmic auditing pipelines should integrate uncertainty‐aware validation matrices that quantify prediction confidence thresholds for high‐stakes therapeutic targets—particularly crucial when modeling conformationally dynamic proteins like viral fusion peptides or allosteric kinase domains. In parallel, a tiered biosecurity classification system requires developers to flag and isolate sensitive protein predictions (e.g., toxin‐receptor interfaces or oncogenic mutation hotspots) through cryptographic proof‐of‐stake mechanisms, ensuring controlled access via blockchain‐verified multi‐signature authentication. To operationalize global equity, a decentralized computing infrastructure could leverage sharded database architectures with region‐specific model distillation. This approach would enable low‐resource institutions to access core AF3 functionalities through lightweight API‐mediated prediction servers while maintaining centralized oversight of sensitive computational workflows.

Collectively, these measures balance innovation acceleration with biosafety imperatives, ensuring AF3 serves as an equitable tool for global drug discovery rather than a source of technological disparity or biological risk. To address regulatory issues, major regulatory agencies, such as the US FDA and the EMA, should revise their New Drug Application (NDA) guidelines to require the submission of detailed “AI model profiles” documenting model architecture, training data composition, uncertainty quantification methods, and blind testing results. These profiles would subject the AI ​​to the same rigorous scrutiny as traditional experimental data. To complement these regulatory requirements, an open “computation‐experiment validation sandbox” platform should be established, funded by national research grants. This platform would provide standardized, independent third‐party experimental validation for models like AF3 on key targets, generating high‐quality benchmark datasets for continuous model improvement. To promote global equity, a “Structural Biology AI Equitable Access Fund” led by organizations such as the WHO should be proposed. This fund would not only support low‐ and middle‐income countries in accessing basic computing resources but, more importantly, support them in conducting localized model fine‐tuning and validation for endemic disease targets, thereby building technological capabilities. Finally, by establishing an AI biosafety group composed of international experts, developing a list of risky proteins, and regularly conducting proactive misuse stress tests on predictive tools, while requiring developers to comply with international transparency reporting protocols and accept global oversight while protecting legitimate research, these proposals collectively constitute a comprehensive action network encompassing regulation, verification, collaboration, empowerment, and oversight.

## 9 Accelerating the Practical Applications of AlphaFold 3

To accelerate the applications of AF3, the primary task is to establish a standardized, multidimensional benchmark set that encompasses not only static structures but also conformational dynamics, weakly interacting interfaces, and multi‐component assembly systems, building on existing benchmarks for predicted complexes. Simultaneously, a hybrid validation protocol needs to be developed and promoted, validating predictions experimentally with methods such as crystallography and cryo‐electron microscopy, while encouraging cross‐validation with molecular dynamics simulations or enhanced sampling methods for low‐confidence or biologically high‐stakes predictions. Computational resource planning at this stage should focus on dedicated, reproducible high‐performance computing for these benchmarking and validation efforts. Then, the focus can shift from static structural analysis to dynamic mechanism research. The key to technological development lies in developing algorithms capable of simulating conformational changes and binding/dissociation pathways in protein‐ligand complexes. This requires deep integration of AF3 predictions with free energy perturbation and dynamic simulations. Furthermore, an iterative prediction–experiment cycle is needed, in which discrepancies between predicted and experimental structures are documented and incorporated into the next generation of training data. Therefore, computing resources need to be scaled up significantly to support large‐scale free energy calculations and periodic model retraining, which will require the coordinated scheduling of heterogeneous computing resources.

Ultimately, this requires the democratization of capabilities and expansion into cutting‐edge areas. On the one hand, lightweight, low‐power versions need to be developed using techniques such as knowledge distillation and model compression, enabling them to run on modest GPU or even CPU‐based laboratory computing resources and promoting equitable global access, since AF3's bottlenecks lie in compute and licensing rather than in access to the model itself. On the other hand, resources should be directed towards more exploratory directions, such as predicting complex chemical modifications, the integration of non‐canonical amino acids, and the assembly of ultra‐large complexes with components like nucleic acids and lipids. The strategic focus of computing resources will shift to building dedicated computing facilities and efficient, shared cloud platforms for biology to support these massive and complex computational tasks.

However, even accurate prediction of protein structures does not guarantee complete success in developing drugs that target specific proteins. The development of methods for producing and synthesizing proteins and chemical compounds is equally important. Designing and developing drugs from scratch remains a highly complex undertaking; while peptides and many soluble proteins can already be produced routinely, membrane proteins, ultra‐large complexes, and heavily modified proteins remain difficult to obtain in the quantities and quality that structural and functional studies require. The ultimate value of AF3 lies in its unprecedented speed and breadth in raising the “starting point” of target identification and validation to a high‐precision structural level. This can significantly reduce “blindness” but does not eliminate the inherent “uncertainty” in drug development (Table 3). It is expected to transform drug discovery from “guessing based on limited structures” to “generating and prioritizing rational hypotheses based on rich structural information.” As these problems are resolved, more antibodies and clinical therapeutics will be developed [28], shaping the drug development market.

**TABLE 3:** Cases of the application of AlphaFold 3.

Case	Application of AF3	Results
Evaluate fatty acid docking to FABPs	Predicted high‐accuracy 3D structures for single proteins	The conformations and positions of FAs docked in FABP3–5 and FABP8 using AF3 showed similarity to the experimental structures [29].
Integrate AF3 with LFIA for detection	Modeled antibody–antigen complexes for immunoassay	The structure of the monoclonal antibody was accurately predicted using AF3 [30].
Assess AF3 in predicting RNA structures	Predicted 3D RNA structures	AF3 was capable of producing atomic models for RNAs containing up to 2000 nts that are free of gross steric clashes [31].
Study Zn^2+^‐mediated inhibition of caspase‐3	Predicted Zn^2+^ binding sites	AF3 and molecular dynamics simulations revealed a mechanism for non‐competitive inhibition [32].
Model intrinsically disordered proteins	Predicted secondary structure in α‐synuclein	AF3 could benefit from stochastic sampling or Monte Carlo simulations for generating an ensemble of structures for intrinsically disordered proteins [33].

## 10 Conclusions

With its ability to predict protein and protein‐complex structures, AF3 holds considerable potential to reshape the DDD pipeline in the AI era. Specifically, it can support the identification and validation of drug targets, assist hit identification and lead optimization, and contribute to the development of precision medicine, thereby improving the efficiency of R&D for innovative therapeutics while reducing attrition in later stages—though the extent of these gains remains to be established in prospective studies. Of particular note is its capability to predict protein‐ligand interactions and certain post‐translational modifications, offering critical insights to improve drug efficacy and selectivity; its handling of conformational diversity remains limited, however, and must be complemented by molecular dynamics simulations. Moreover, integrating AF3 into current personalized medicine frameworks may move the field toward more effective and efficient healthcare, provided that the ethical and governance questions discussed above are addressed. Despite the technical challenges and ethical considerations associated with AF3, its emergence represents a meaningful step forward in the DDD landscape. As this technology continues to evolve, AF3 and its future iterations will likely accelerate the creation of new medicines.

## Author Contributions

**Songlin He**: conceptualisation, visualisation, writing − original draft– review and editing. **Liangbin Zhou**: conceptualisation, writing − original draft– review and editing. **Xiaoshuai Wang**: writing – review and editing. **Chenzhong Li**: writing – review and editing. **Cheng Jiang**: writing – review and editing. **Rocky S. Tuan**: conceptualisation, supervision, writing – review and editing, funding acquisition. **Zhong Alan Li**: conceptualisation, supervision, writing – review and editing, funding acquisition.

## Conflicts of Interest

The authors declare no conflicts of interest.

## Data Availability

Not applicable.
